# Singapore's HIV disclosure law in the context of progress towards the 90-90-90 goals: A call for greater action in the Western Pacific

**DOI:** 10.1016/j.lanwpc.2022.100588

**Published:** 2022-09-07

**Authors:** Rayner Kay Jin Tan, Alex R. Cook

**Affiliations:** a University of North Carolina Project-China, Guangzhou, Guangdong, China; b Saw Swee Hock School of Public Health, National University of Singapore and National University Health System, 12 Science Drive 2 MD1 Tahir Foundation Building Level 10 Singapore 117549, Singapore

Singapore's courts sentenced a 48-year-old man in June 2022 to a year's imprisonment for failing to tell two people he had sex with that he was living with HIV, an offence under the city state's Infectious Diseases Act. The ruling came in spite of the man in question having medical evidence of being successfully virologically suppressed through antiretroviral therapy and thus at no risk of transmitting HIV to his partners.

Unequivocal evidence from studies in multiple countries, spanning several partnership types, show that people who have HIV and have achieved viral suppression through treatment cannot transmit HIV through sex.^1^, ^2^, ^3^ This forms the basis of treatment as prevention and is one of the key reasons why progress towards the Joint United Nations Programme on HIV/AIDS (UNAIDS) 90-90-90 goals offers hope of an end to HIV transmission in the coming years.

In Singapore, where substantial progress towards the 90-90-90 goals has been made—91% of people with diagnosed HIV are getting treatment, and 91% of those on treatment have non-detectable viral loads^4^—there is now a sizable number of people who face jail for not communicating that they have what, in essence, has been rendered a non-communicable disease. This situation is indicative that advances in science are outpacing advances in the law.

The criminal provision in question was passed in 1992, to deter “irresponsible and dangerous” behaviour from people living with HIV, and had the aim of preventing onward transmission of HIV to others.^5^ At the time, mandatory disclosure of one's HIV status to sexual partners could be justified as medication would at best delay the onset of late-stage infection, and the risk of transmission to others was very real. Today, however, medications enable people living with HIV to have life expectancies that are essentially the same as those who do not have HIV.

At face level, a law imposing responsibility on those living with HIV to disclose their infection status to their sexual partners, even if there is no longer a risk of transmission, seems benign. There are, however, reasons to believe disclosure laws are not favourable for HIV prevention, beyond the simple fact that, in Singapore, almost 9000 people have nevertheless been diagnosed with HIV since the provision came into play in the early 1990s.^6^ An expert consensus statement on the science of HIV in the context of criminal law published in 2018 underscores this point.^7^

First, Singapore does not have anti-discrimination employment protections for people living with HIV, and there remains a heavy social stigma attached to HIV. Thus by obligating disclosure, the law exposes people living with HIV to risk without commensurately protecting them from the potential harms that result. Second, the law creates a disincentive to test for HIV, because a positive diagnosis would impose a legal responsibility on them to disclose their HIV status to others. This harms Singapore's progress towards the final goal the UN has set for HIV elimination, namely that more than 90% of people living with HIV know their status. Third, it further stigmatises HIV, which feeds back into the loop that disincentivises testing, getting to know one's HIV status, seeking out treatment, and reaching a viral load that is undetectable, hence preventing transmission.

According to the HIV Justice Network, by the end of 2021, many countries in the Western Pacific region continue to retain HIV-specific laws.^8^ These include provisions in the criminal code, provisions in HIV-specific laws, or provisions that single out people living with HIV for harsher sentences. In lieu of HIV-specific laws, several countries have also applied general laws to HIV criminalisation cases.^8^ These all go against recommendations by UNAIDS, which suggest avoiding overly broad use of the law to criminalise HIV, and limiting punishment to rare cases of intentional and proven HIV transmission with malicious intent.^9^

Nevertheless, we are seeing positive developments take place in some jurisdictions that have updated analogous laws to reflect the medical advances in HIV treatments. For example, Taipei recently amended its ‘criteria for unsafe sexual behaviour’ under its HIV criminalisation laws to recognise that individuals who are virally suppressed should not be considered under the criteria of having unsafe sexual behaviour.^10^ The last three decades have witnessed remarkable changes in what it means to live with HIV, thanks to major scientific and medical advances. It may be timely to reassess whether laws such as Singapore's remain aligned with the current reality of HIV as a chronic health condition controllable through medication.

## Contributors

RKJT and ARC conceptualised this correspondence, drafted the initial manuscript, and approved the final version of the manuscript.

## Declaration of interests

The authors declare no conflict of interest.
